# Sizing the Frozen Elephant Trunk Based on Aortic Pathology and the Importance of Pre-Operative Imaging

**DOI:** 10.3390/jcm12216836

**Published:** 2023-10-29

**Authors:** Marinos Koulouroudias, Konstantinos Velissarios, John Kokotsakis, Dimitrios E. Magouliotis, Pantelis Tsipas, Arian Arjomandi Rad, Alessandro Viviano, Antonios Kourliouros, Thanos Athanasiou

**Affiliations:** 1 Department of Cardiac Surgery, Nottingham University Hospitals NHS Trust, Nottingham NG5 1PB, UK; marinosk@doctors.org.uk; 2 Medical Horizons, 15562 Cholargos, Greece; kvelissarios@medicalhorizons.gr; 3 Department of Cardiac Surgery, Evangelismos Hospital, 11527 Athens, Greece; jkokotsakis@gmail.com (J.K.); tsipasp@yahoo.gr (P.T.); 4 Department of Cardiothoracic Surgery, Larissa General University Hospital, 41334 Larissa, Greece; dimitrios.magouliotis.18@ucl.ac.uk; 5 Department of Cardiac Surgery, Oxford University Hospitals, Oxford OX3 9DU, UK; arian.arjomandirad@gmail.com (A.A.R.); antonios.kourliouros@ouh.nhs.uk (A.K.); 6 Department of Cardiac Surgery, Hammersmith Hospital, Imperial College Healthcare NHS Trust, London W2 1NY, UK; alessandro.viviano@nhs.net; 7 Department of Surgery and Cancer, Imperial College London, London W2 1NY, UK

**Keywords:** aortic dissection, frozen elephant trunk, aortic aneurysm

## Abstract

The frozen elephant trunk is a formidable tool for the aortovascular surgeon. An appreciation of how to size the graft in different pathologies is key in achieving optimal results. Herein, we demonstrate worked examples of how imaging can be used to plan for a frozen elephant trunk and discuss the nuisances and uncertainties of sizing using three index cases: Type A aortic dissection, distal thoracic aortic aneurysm and chronic dissection.

## 1. Introduction

The management of complex arch and descending thoracic aortic pathologies presents some of the most formidable operative challenges in aortovascular surgery. The initial development of the classical elephant trunk by Borst [1] and the subsequent modification into the Frozen Elephant Trunk (FET) [2] has opened up the possibility of simplifying a two-stage approach to the complex arch/descending thoracic aorta or even treating the whole aorta in a single operation (Table 1, indications adapted from EACTS position paper [3]). With the wide availability of off-the-shelf FET prostheses (Table 2, Thoraflex [4] and E-vita [5] characteristics), there is growing interest in how to optimise case selection, operative technique and patient outcomes. One of the key features of the FET that makes it an attractive solution is its customisability and versatility to adapt to anatomical and pathological variation.

However, to make the most of the FET’s versatility, the surgical team needs to carefully interrogate the pre-operative Computed Tomography Angiogram (CTA) and individualise the approach for each patient based on pathology, body habitus and aortic configuration.

Here, we will discuss the central role of using imaging to guide sizing of the FET based on underlying pathology using three examples of using the FET in acute aortic dissection, chronic dissection and aortic aneurysms.

## 2. Materials and Methods

Key demographic data, clinical characteristics and operative reports were analysed across our case series of frozen elephant trunk implantations (69 cases from November 2007 to June 2023) to identify three indicative cases, for acute type A dissection, degenerative aneurysm and chronic dissection.

All imaging were anonymised and patients consented to imaging being used for research and subsequent publication.

Key images are presented with a 3D reconstruction of the whole aorta pre- and post-operatively with demonstration of cross-section analysis perpendicular to the aorta at different levels. We provide key cross-sections of the descending thoracic aorta at the level of the elephant trunk portion and distal to the elephant trunk, demonstrating pertinent diameters of the aorta and true/false lumen as indicated.

All analyses were performed using the 3mensio Vascular software (version 10.3, Pie Medical Imaging B.V., Maastricht, The Netherlands)

## 3. FET Type and Size Selection According to Pathology

There are many proposed methods and a lack of consensus on how to size the FET. Various protocols have been suggested by multiple experienced aortic teams (e.g., Bologna [6], Essen [7], Hanover [8] etc.).

Here, we demonstrate how imaging can be used to size the FET based on our experience.

In all cases, we start the analysis by creating a centreline from the aortic root to the lowest possible level (depending on the CTA coverage section of the body) that is always parallel to the vessel, thus giving the ability to obtain true cross-sections perpendicular to the aorta at all levels. Perimeter-derived diameters are measured in several levels of the ascending aorta, aortic arch and thoracoabdominal aorta.

### 3.1. Length of the FET

The key concern is to minimise the risk of spinal cord ischaemia without compromising the outcome of treatment.

We routinely size the FET endopart length to go around the curvature of the distal arch but not more than the level of T7, especially if there are patent intercostal arteries of significant size.

Lengths are always measured in vessel stretch display.

Additional to the centreline, an outer curve line is also drawn from the intended FET distal anastomosis level to distal descending aorta to estimate the anticipated distal landing zone more accurately.

#### 3.1.1. Type A Acute Aortic Dissection

Size: Measurements of true as well as the total aortic diameter (both true and false lumen) are taken at the level of the left atrium to determine the graft size.

**Figure 1:**
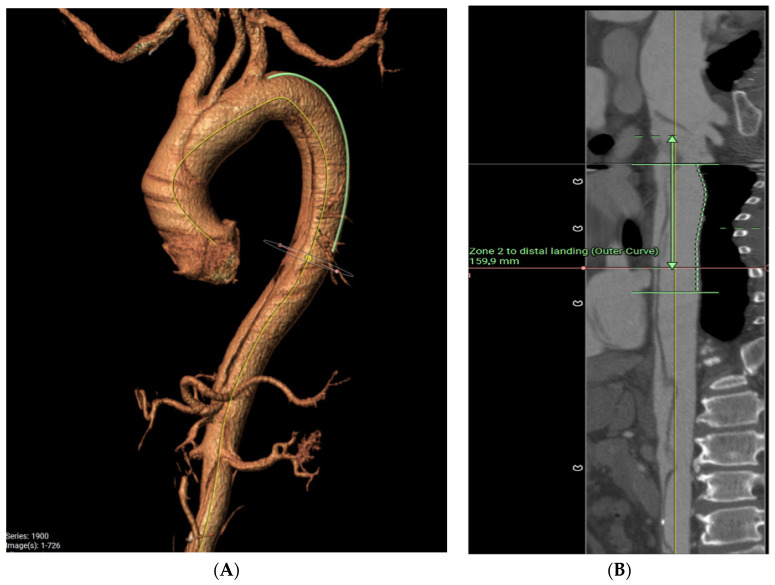

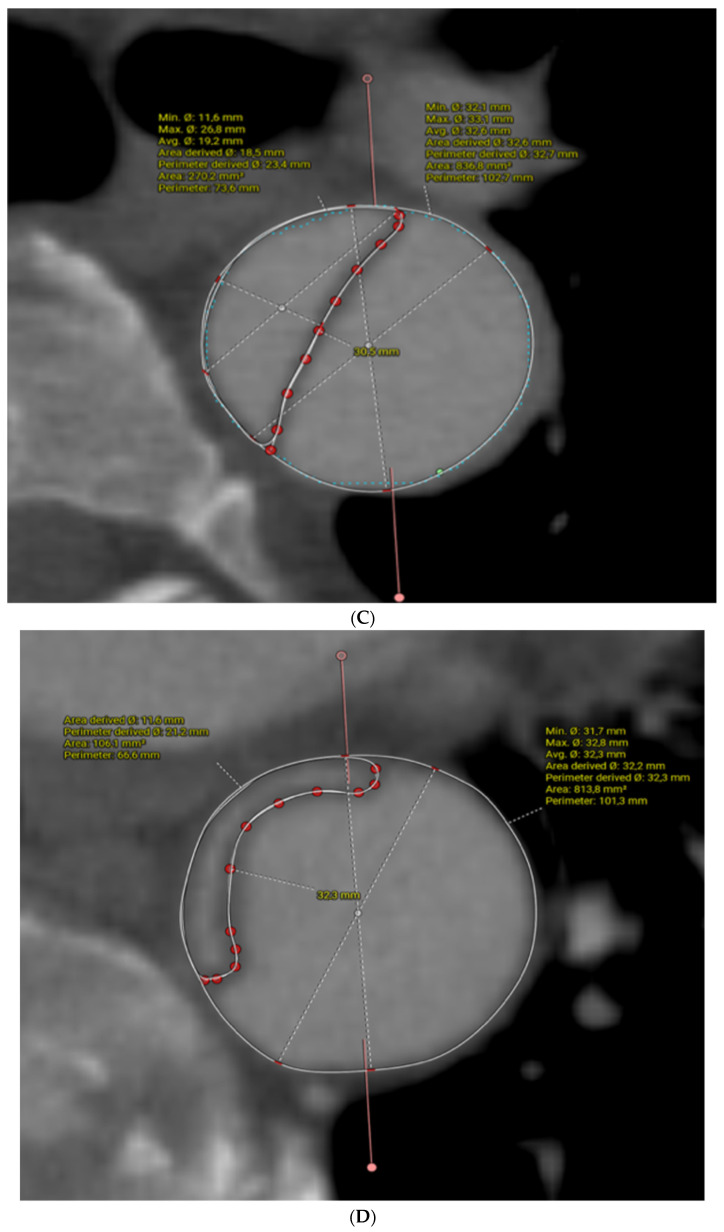
Pre-operative CT angiogram analysis for sizing a FET in acute type A aortic dissection, using centreline analysis and multiplanar reconstruction. Yellow line is the centreline and green marks the outer curve in (**A**). (**B**) is the vessel stretch display where the distal landing zone indicated by the arrow is estimated. (**C**,**D**) demonstrate the measurement of the dissection septum and aortic diameter. See full text for further explanation.

In this case, a pre-operative 3D reconstruction with centreline analysis and multiplanar reconstruction of a De Bakey I dissection is shown in Figure 1. The yellow line represents the centreline and green the outer curve line. A perimeter is drawn around the areas of interest, both the true lumen as well as the whole aorta and the perimeter-derived diameter is taken into consideration. The length of the septum is also measured via a curved line. The average of both the septum length and total aortic diameter is used for graft diameter size and the anticipated distal landing is estimated on the vessel stretch display (Figure 1B).

Figure 1C,D demonstrate the dissection septum and diameters at levels above and below the distal FET endopart, respectively.

**Figure 2:**
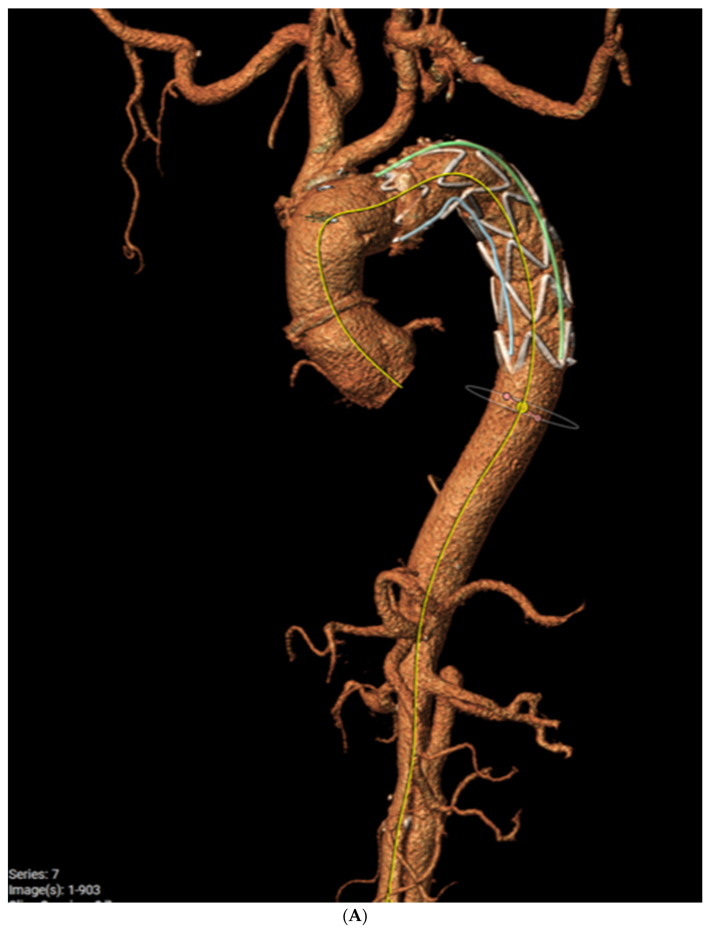

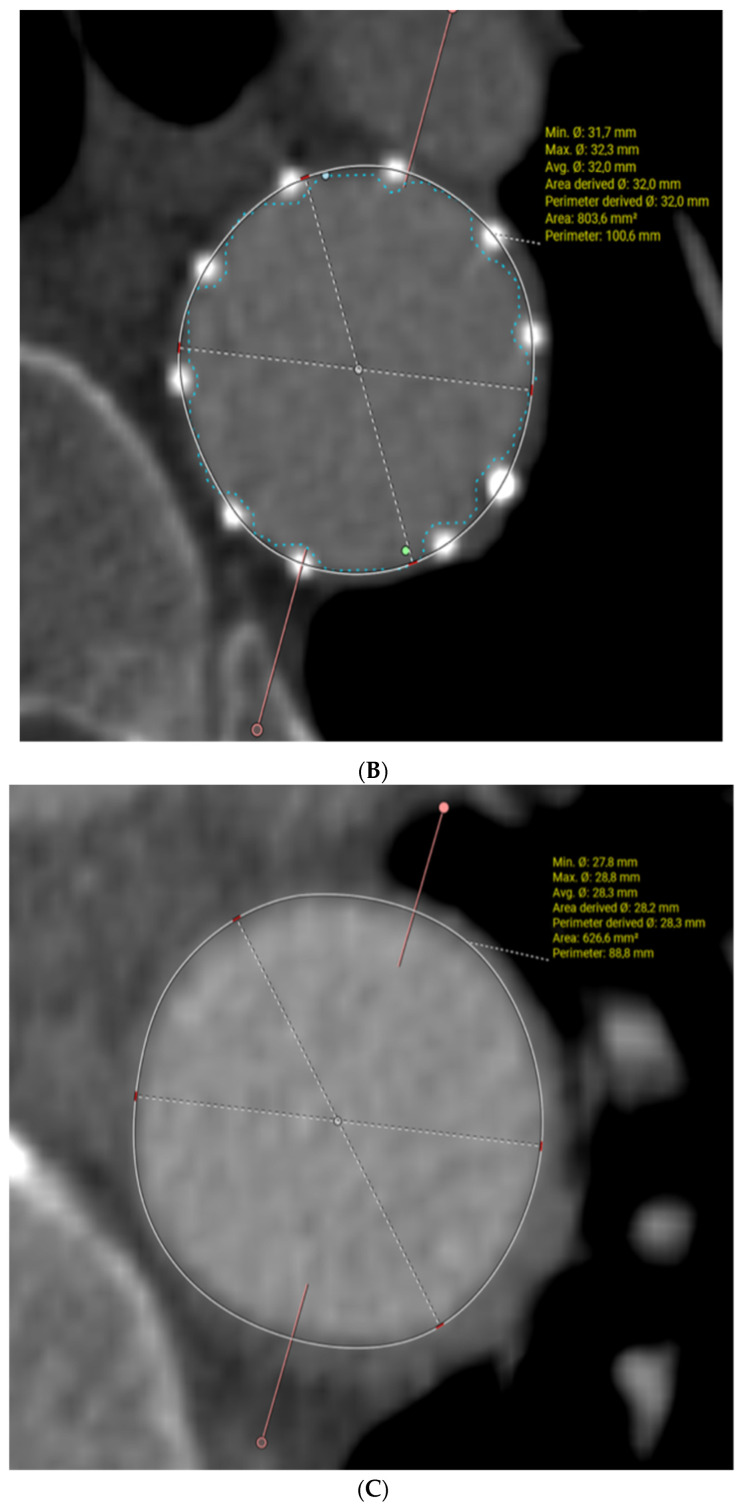
Post-op evaluation of the FET for acute aortic dissection, (**A**) demonstrates 3D reconstruction of the post-FET appearance of the aorta, (**B**) demonstrates the full expanded stent graft in the descending aorta and (**C**) demonstrates the resolution of the false lumen distal to the stent graft.

In Figure 2, a post-FET, 3D multiplanar reconstruction with centreline analysis is shown (Figure 2A). This demonstrates the full expansion of the endopart according to a pre-operative measured graft size of 32 mm (Figure 2B) and fully expanded true lumen below the FET level with restoration of a normal diameter at an average of 28mm and complete disappearance of the false lumen (complete aortic remodelling) (Figure 2C). However, dissection persists in the abdominal aorta with perfusion of all abdominal branches due to residual communicating tears. 

Other imaging considerations: Inspect presence and location of re-entry tears in thoracic and abdominal aorta to avoid issues of FET malposition in false lumen and malperfusion of distal branches.

#### 3.1.2. Chronic Aortic Dissection

Size: We size chronic dissections based on the true lumen perimeter-derived diameter as the rest of the aorta and the septum between the true and false lumen can be very stiff/non-compliant. Measurements are taken at 2 cm intervals on the descending aorta and the graft is sized at the location where we expect the endopart to be deployed.

**Figure 3:**
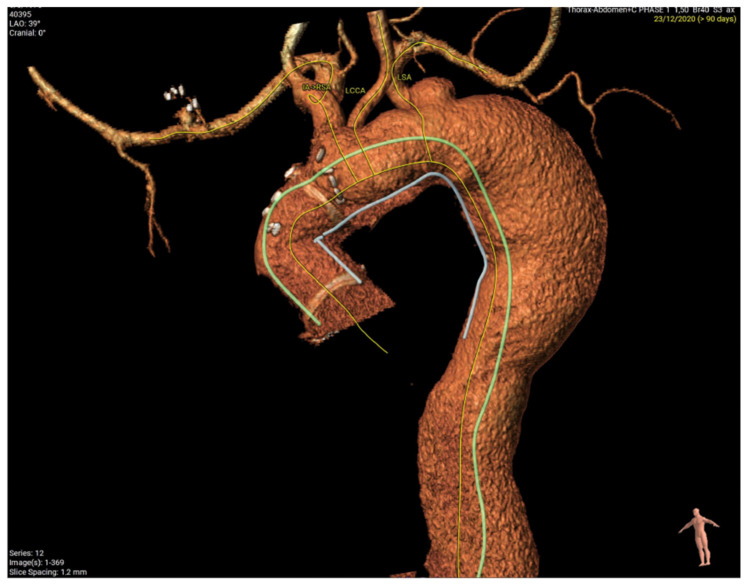
Young patient with previous ascending aorta replacement after acute type A dissection presenting with distal anastomosis new entry (DANE) tear and big communicating tear at distal arch with rapid expansion of his descending thoracic aorta one year post-operatively.

**Figure 4:**
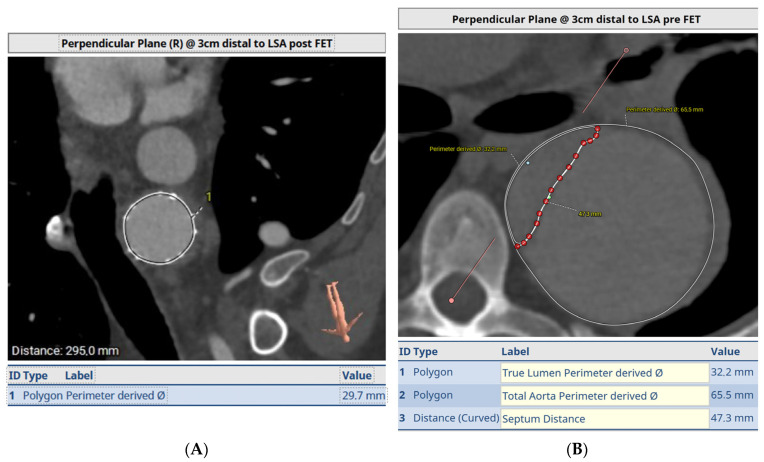
Sizing of the FET based on the true lumen (**A**) and post-operative image of the same site (**B**) demonstrating full re-expansion of the true lumen.

**Figure 5:**
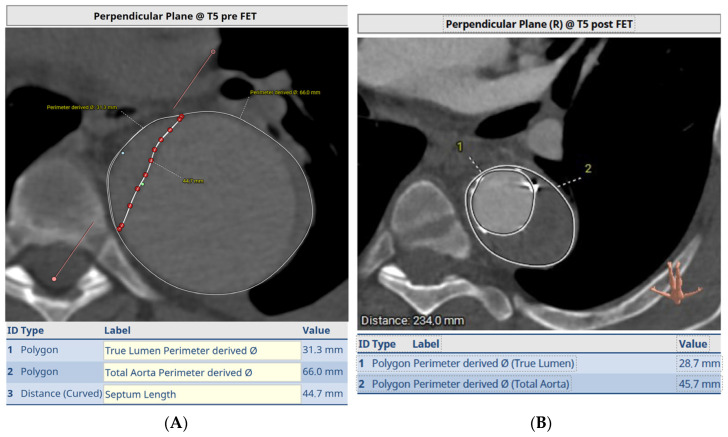
Pre-op (**A**) and post-FET (**B**) at level of T5 demonstrating expansion and ressurization of the true lumen.

In this case, (Figure 3), a young patient with previous ascending aorta replacement for acute type A dissection presented with a distal anastomosis new entry (DANE) tear and a big communicating tear at the distal arch with rapid expansion of his descending thoracic aorta one year post-operatively. Figure 4 shows sizing of the FET based on the true lumen (Figure 4A) and a post-operative image of the same site demonstrating full re-expansion of the true lumen. Figure 5 shows pre-op (Figure 5A) and post-FET (Figure 5B) reconstructions at the level of T5 demonstrating expansion and pressurisation of the true lumen. Due to the ongoing perfusion of the false lumen and continuation of dilatation of the descending aorta, a second-stage endovascular aneurysm repair was required and facilitated by the FET.

Other imaging considerations: Careful inspection should be made to see if the false lumen is supplying intercostal/visceral arteries and whether distal re-entry tears are present. In the absence of distal re-entry and dependence of perfusion on the false lumen only, implantation of the FET might be catastrophic.

#### 3.1.3. Thoracic Aortic Aneurysms

Size: In chronic thoracic aortic aneurysms, we suggest a 10–20% oversize and aim for a landing zone of more than 30 mm. The key consideration in aneurysmal disease is to minimise both the chance of endoleak as well as avoid occlusion of lower intercoastal arteries.

**Figure 6:**
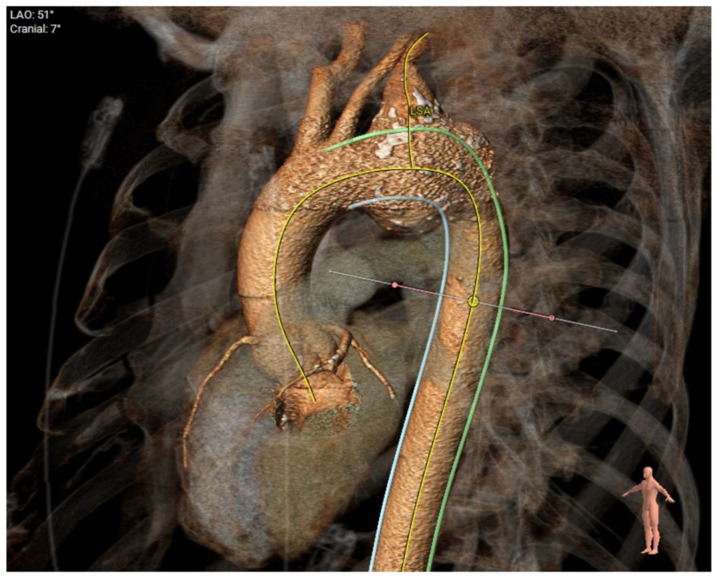
Degenerative aneurysm of the distal arch/proximal descending thoracic aorta involving the origin of the left subclavian artery.

**Figure 7:**
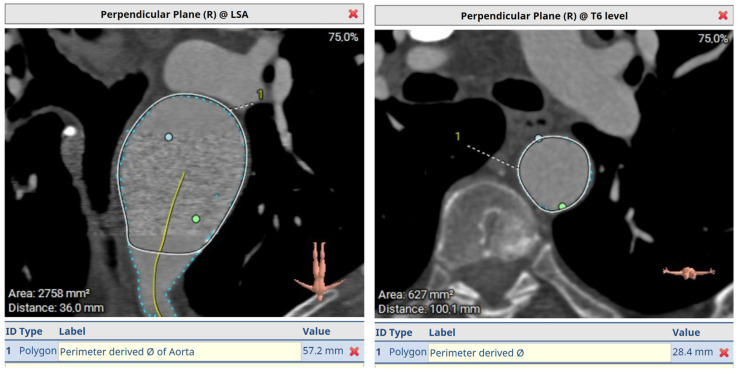
A pre-op aortic diameter at left subclavian level of 57.2 mm and 28.4 mm in estimated distal landing area (T6 level) which dictated the implantation of a 33 mm graft. Post-FET reconstruction in Figure 8 demonstrates shrinkage of aneurysm sack at LSA and sealing of the aorta at T6.

**Figure 8:**
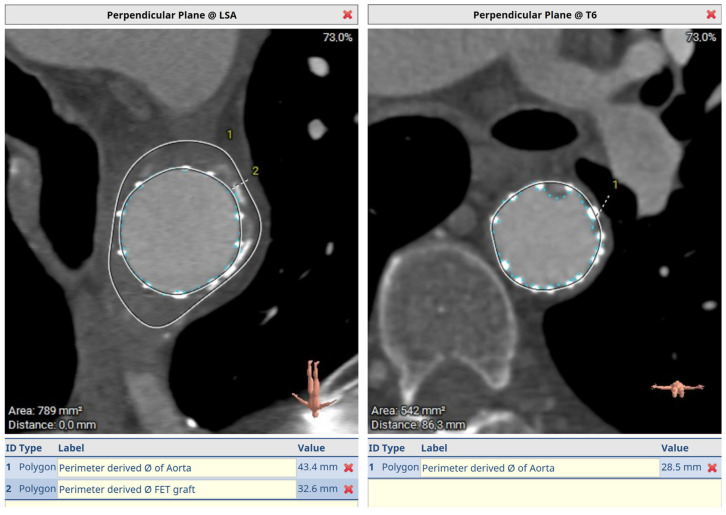
Post-FET perpendicular plane at LSA with aneurysm shrinkage and distal sealing at T6.

Figure 6 demonstrates a degenerative aneurysm of the distal arch/proximal descending thoracic aorta involving the origin of the left subclavian artery. Figure 7 shows a pre-op aortic diameter at the left subclavian level of 57.2 mm and 28.4 mm in the estimated distal landing area (T6 level) which dictated the implantation of a 33 mm graft. Post-FET reconstruction in Figure 8 demonstrates shrinkage of the aneurysm sack at LSA and sealing of the aorta at T6.

**Figure 9:**
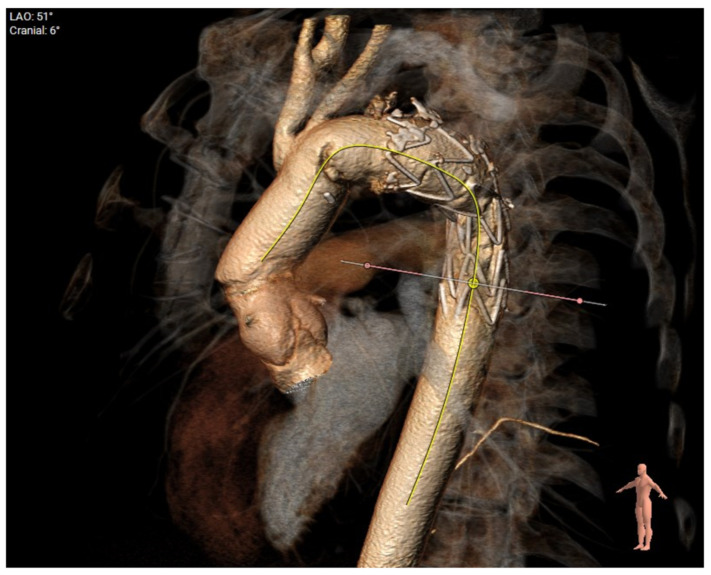
Post-op 3D reconstruction of FET for aneurysmal disease. Coverage of aneurysm at origin of subclavian demonstrating oversize and a shorter landing zone of around 30 mm.

Finally, Figure 9 demonstrates the post-op 3D reconstruction of FET for aneurysmal disease.

## 4. Discussion

Our report demonstrates the use of pre-operative aortic imaging and downstream reconstruction to size the frozen elephant trunk, in advance of any operation.

Due to the lack of consensus as to how to size a FET, we present the key considerations presented in the literature, as well as a potential generic algorithm for sizing in different pathologies.

Finally, we summarise our recommendations for future research and guideline development in this evolving field.

### 4.1. The Use of CT Angiography Is Central to Planning for a FET

We feel that a high-quality, ECG-gated CTA from the circle of Willis and down to the femoral arteries should be mandatory in all cases where aortic interventions are contemplated.

Downstream analysis should use dedicated software for aortic centreline measurements and multiplanar reconstructions for ascertaining aortic lengths and orthogonal diameters. Specialised vascular imaging software can be used that provides dedicated workflows for high-quality and reproducible measurements, such as our demonstration of the use of 3mensio.

Appropriate sizing for the diameter and length of the stent graft is key to:

(a) Maximise true lumen perfusion;
(b) Minimise spinal cord injury;
(c) Minimise risk of type Ib endoleak;
(d) Mitigate the risk of future re-interventions.

We feel that the key components to examine in a pre-FET CTA should include the diameters of the ascending, arch and DTA at the level of the pulmonary artery and/or left atrium, the diameter of arch vessels and the distance between the arch vessels/landing zones (especially between left common carotid and left subclavian artery).

Furthermore, one should make note of the dominance, origin and patency of the vertebral artery and presence and location of the artery of Adamkiewicz.

In cases of aortic dissection, the location and extent of proximal aortic communicating tears and their relation to the left subclavian origin, as well as distal communicating tears, should be noted. The patency of the true lumen, presence of radiological malperfusion and dependence of peripheral vascular beds on false lumen perfusion are also key considerations.

Other, generic considerations such as assessment of cannulation sites for cardiopulmonary bypass, aortic wall quality (calcification and atheroma), etc. are also important in achieving successful outcomes.

### 4.2. Sizing for Acute Aortic Dissections

Maximising false lumen thrombosis is a key consideration in optimising long-term outcomes beyond the early post-operative period. A meta-analysis from Li et al. in 2016 demonstrates the key role of the residual patent false lumen in long-term mortality, as well as aortic re-intervention (HR 5.43, CI 2.95–9.99, *p* < 0.0001) [9]. At the same time, the analysis from Fattouch et al. [10] highlights the stark contrast in mortality and risk of re-intervention when the false lumen remains patent (89.8 vs. 59.8% 10 year survival and 94.2 vs. 63.7% freedom from distal re-intervention). Patency of the false lumen has been reported to range from around 5–25% post-FET at mid-term with most case series reporting complete thrombosis in long-term follow-up [11,12]. Yet, the natural history of false lumen thrombosis can be unpredictable in its timing and evolution.

Sizing to the total aortic diameter allows the still compliant dissection flap (septum) to fully re-expand and minimise false lumen flow [13,14]. This compliance of the dissection flap in acute dissections might mitigate the risk of aortic injury even when sizing to total diameter. A study by Hoffman and colleagues [13] demonstrates that sizing to total aortic diameter can be safe, with no stent perforation/aortic injuries at least at mid-term follow-up.

On the contrary, there can be some apprehension that sizing to total diameter can risk leading to improper expansion of the stent graft, aortic injury and dSINEs [15]. As such, some suggest to size the FET based on true lumen diameter and keep patients on optimal medical therapy, surveillance and potentially offering completion TEVAR to achieve false lumen thrombosis whilst mitigating risk of dSINEs and aortic injury [13].

When considering the risk of dSINEs, it is important to remember that they can be associated with up to 25% risk of mortality post-acute dissection repairs and are much more relevant post-FET (6% incidence post-TEVAR, 25% at 3 years post-FET) [15,16]. Hence, minimising the risk of this common and morbid complication is a key consideration.

Complicating matters further, there are no established clinical risk factors for post-FET dSINEs, and oversizing does not appear to always be a key pathway leading to dSINEs. Proposed mechanisms include short grafts, with a sharp angle between the graft–lumen interface and the presence of connective tissue disease [15,17].

With the lack of clear evidence, we feel that in most cases of acute dissections, sizing to the total aortic diameter with no oversizing is acceptable to maximise FL thrombosis and achieve optimal remodelling and long-term positive outcomes without an excessive risk of injury to the aortic wall or intima.

### 4.3. Sizing in Chronic Dissections

The use of the elephant trunk technique can help in excluding false lumen dilation evident in chronic dissection, promoting aortic remodelling and minimising the risk for further re-interventions.

There is some variability in sizing practices at major aortic institutions for chronic dissections with the Bologna group [6] sizing to the maximal true lumen diameter, the Hannover group using the largest possible grafts by sizing to the total aortic diameter [18] and the Essen group undersizing or even using the smallest possible grafts [7]. Despite this variability, all these aortic centres report comparable outcomes for mortality and distal re-interventions.

The main concern in sizing for a chronic dissection is that the flap becomes more inelastic, as suggested by computational models and clinical observation [19]. As such, oversizing could lead to dSINEs and pseudo-coarctation or even aortic rupture.

This concern is mostly extrapolated from the TEVAR literature [20], where chronicity of dissection and the presence of oversizing is a strong factor predisposing to distal new entry tears, however, this was not consistently replicated in studies looking at the FET (perhaps owing to a smaller number of patients).

Finally, sizing a FET for chronic dissection also needs a careful examination of the CT aortogram to assess whether false lumen flow is responsible for distal vascular bed perfusion and whether any distal communication tears are providing flow to key intercostal, visceral or peripheral vascular beds. Excluding the false lumen and inducing false lumen thrombosis in this setting can lead to catastrophic malperfusion.

To mitigate the risk of potential aortic injury, we suggest (in accordance with multiple reports and reviews [3,7,18]) to use the smallest stent graft size possible and not larger than the true lumen diameter.

### 4.4. Sizing the FET for Aneurysmal Disease—Minimising Endoleak

Utilising the FET for aneurysmal disease, one needs to balance the risk of type 1b endoleak by appropriately oversizing the graft and distal sealing zone length versus the risks of spinal cord injury, graft crumpling and aortic disruption.

Kandola et al. in a retrospective analysis of FET use in aneurysms demonstrate that the majority of endoleaks occurred with <10% oversizing and <30 mm distal seal zone and virtually no endoleaks when oversizing was achieved by >10% and a >30 mm distal landing zone [21]. Furthermore, with more aggressive oversizing, no spinal cord injury was observed and no adverse events including aortic injury. Similar outcomes were also reported by Chu et al. with a distal seal zone of 30–40 mm [22]. These findings also echo the TEVAR community recommendations of distal landing zones of >20 mm and >10% oversizing [23,24].

We recommend, in the absence of larger-scale registries, to adopt 10–20% oversizing and an at least 30 mm distal seal zone.

### 4.5. Spinal Cord Injury—Achieving Appropriate Coverage whilst Minimising Risk

Paralysis or paraparesis post-FET is a devastating complication that compromises long-term outcomes and has a detrimental effect on quality of life. This can occur in up to 25% of patients. The Preventza meta-analysis identified a subgroup of patients in whom the graft length was >15 cm and covered beyond T8 that had a risk of spinal injury of 11.6% (95% CI 6.1–21.1%) versus 2.5% (95% CI 1.5–4.0%) where a coverage <T8 and <15 cm was achieved [25].

On the contrary, there is some evidence that it is safe to achieve a longer graft coverage with up to 12 segmental arteries being sacrificed without substantial increased spinal injury risk [26]. As such, it has been suggested that planning for a landing zone at T10–12 can be safely achieved and might even allow for better collateralisation of the spinal cord in the inter-period prior to completion of TEVAR in chronic type B dissections/aneurysms.

However, we err on the side of caution and aim for coverage up to T7 based on the subgroup analysis from the largest meta-analysis on the topic.

### 4.6. The Future Role of Artificial Intelligence in Streamlining Planning for the FET

Timely diagnosis and intervention in acute aortic syndromes and large aneurysms are a key priority for healthcare systems due to their associated morbidity and mortality. However, diagnosis can be challenging due to the rarity of the condition and its variable presentation, including mimicking other emergencies such as stroke and myocardial infarction.

Fast-tracking the identification of patients with high pre-test probability for aortic emergencies to obtain an appropriate CT angiogram is paramount in minimising the risk of misdiagnosis [27].

The comprehensive interpretation and downstream processing of aortic imaging in planning for a FET procedure can be time-consuming and a limiting factor in its uptake in the setting of acute presentations. Development of machine-learning algorithms that can rapidly size the aorta in its whole length and identify the true and false lumen trajectories and communications and the extent of false lumen thrombosis will be a highly anticipated innovation. There is growing literature on the use of artificial intelligence in segmenting the aorta to obtain haemodynamics and dimensions with encouraging results with good correlation with conventionally obtained measurements [28,29].

Integration of artificial intelligence in expeditious, simple and accurate planning for arch/DTA interventions could be a strong driver in expanding the use of FET in the years to come.

### 4.7. Limitations and Future Directions in Defining the Gold Standard

It is of importance to highlight that there is still a lack of overall consensus on how to size the FET in the absence of large, well-designed registries aiming to address this question.

This is reflected in the variable practice base as observed in a recent survey [30] of multiple large aortic units in North America, the EU and Asia demonstrating high heterogeneity of how units size the FET. This is further evidenced by the lack of robust sizing recommendations from the EACTS position paper on the use of the frozen elephant trunk.

Existing data come from small institutional studies of heterogeneous cohorts (both anatomically and pathologically) with medium-term follow-up or small numbers of patients under long-term follow-up.

Furthermore, the effects of sizing on hard clinical outcomes such as re-intervention, spinal injury and mortality are still unclear.

We suggest that large-scale, international registries need to be established with a vision to clarify how to best optimise sizing the FET and achieving optimal outcomes by:

1. Representing the whole spectrum of pathologies treatable by FET;
2. Capturing all commercially available stent grafts;
3. Standardised nomenclature and definitions of how the stent grafts have been sized (i.e., where the TL or total diameter measurements have been taken on the CT scans);
4. Long-term outcomes reported, with standardised definitions for both aortic adverse outcomes and clinical outcomes.

A clear statistical plan should be formulated to, as far as possible, clarify the relationship of graft sizing with false lumen thrombosis and subsequently the relationship of false lumen thrombosis with outcomes. Finally, the relationship between distal extent of the graft with disease progression versus spinal cord injury should be examined.

This should inform prospective studies to validate the retrospective findings, as well as set the scene for mechanistic/exploratory investigations on the mechanical characteristics of individual stent grafts and aorta–graft mechanical interactions.

Lastly, our recommendations do not take into consideration the rare but important subset of patients with non-atherosclerotic arch pathology (Takayasu’s arteritis, fibromuscular dysplasia, etc.) or the patients presenting with arch dissection/aneurysms in the peri-partum period [31,32]. Large, collaborative retrospective studies should offer granularity in elucidating the appropriate use and sizing of FET in rarer but important subsets of patients.

Only then can we make more robust recommendations and set the gold standard in how to optimise FET sizing for achieving optimal outcomes across the spectrum of aortic diseases.

**Figure 10:**
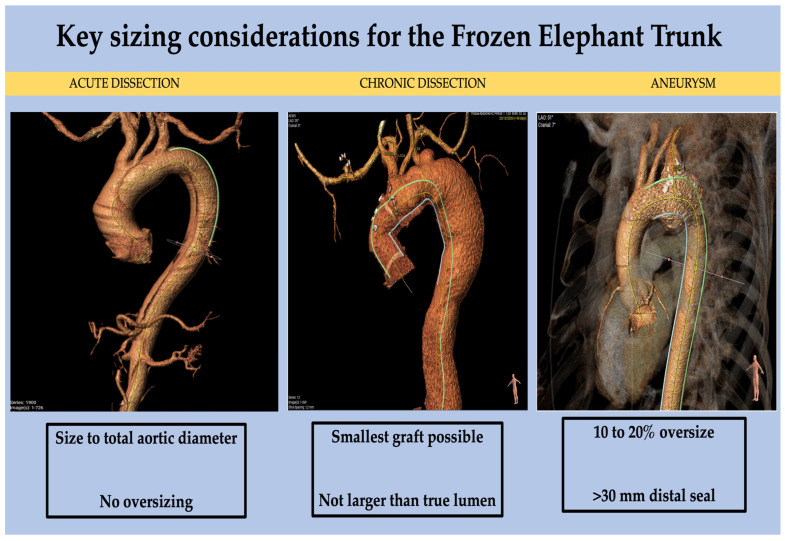
Central illustration: Key sizing considerations, take-home messages.

In the absence of robust data, we offer a summary of our recommendations for sizing based on current based knowledge and experience (Figure 10, central illustration).

## 5. Conclusions

We demonstrate three key cases that highlight the importance of careful pre-operative examination of the CT aortogram in planning for a successful FET across the spectrum of aortic diseases.

## Figures and Tables

**Table 1:** Indications for frozen elephant trunk, adapted from EACTS position statement.

Type A Dissection	Primary entry in distal arch/proximal descending and distal malperfusion or to prevent distal malperfusion. To prevent future aneurysmal dilatation of distal aorta
Complicated Type B Dissection	Can be considered in complex type B where primary TEVAR is not feasible/high risk of retrograde type A
DTA/Thoracoabdominal Aneurysm	Can be considered where there is extensive disease and a second stage to address distal aortic segments can be anticipated
Chronic Dissection	Can be considered to treat post-dissection aneurysmal dilatation of arch and distal thoracic aorta

**Table 2:** Range of sizes and manufacturer recommendations in oversizing/landing zones for Thoraflex and E-vita grafts.

	Thoraflex	E-Vita
Diameter	24–40 mm	20–40 mm
Length	100–150 mm	120–180 mm
Oversizing	15–25% in aneurysmal disease	10–20% in aneurysmal disease
Landing zones	>40 mm in aneurysmal disease	>25–33 mm in aneurysmal disease depending on aortic diameter

## Data Availability

Data presented in this study are available to be shared from the investigators upon reasonable request.
